# A Relation for Nanodroplet Diffusion on Smooth Surfaces

**DOI:** 10.1038/srep26488

**Published:** 2016-05-24

**Authors:** Chu Li, Jizu Huang, Zhigang Li

**Affiliations:** 1 Department of Mechanical and Aerospace Engineering, The Hong Kong University of Science and Technology, Clear Water Bay, Kowloon, Hong Kong; 2 Institute of Computational Mathematics and Scientific/Engineering Computing, Academy of Mathematics and Systems Science, Chinese Academy of Sciences, Beijing 100190, China

## Abstract

In this work, we study the diffusion of nanodroplets on smooth surfaces through molecular dynamics (MD) simulations and theoretical analyses. Molecular dynamics simulations show that nanodroplet surface diffusion is different from that of single molecules and solid particles. The dependence of nanodroplet diffusion coefficient on temperature undergoes a transition from linear to nonlinear as the surface wettability is weakened due to the coupling of temperature and surface energy. We also develop a simple relation for the diffusion coefficient by using the contact angle and contact radius of the droplet. It works well for a wide range of surface wettabilities and different sized nanodroplets, as confirmed by MD simulations.

The dynamics of nanodroplets on surfaces is of great importance in a variety of areas, including the thermal management of micro-electronic devices^1^,^2^, fabrication of self-cleaning surfaces^3^,^4^, nanomaterial synthesis^5^,^6^,^7^, and the design of miniaturized chemical reactors^8^,^9^. In many applications, the diffusivity of nanodroplets plays a critical role because it determines the coalescence and growth rates of droplets on surfaces, which are essential for water harvesting through surface condensation and droplet-based heat dissipation techniques^10^,^11^,^12^,^13^. Understanding the surface diffusion of nanodroplets is also critical for non-mechanical droplet movement control, which is affected by surface properties^10^,^14^,^15^. Unfortunately, nanodroplet surface diffusion is rather complex and is not well understood because it strongly depends on surface properties and temperature, which change the shape (e.g. contact angle) of the droplets.

Previous work on surface diffusion is mainly concerned with the random motion of single molecules, clusters, and solid nanoparticles^16^,^17^,^18^,^19^,^20^,^21^,^22^,^23^,^24^. For single molecules, theoretical analyses can be achieved by treating their motions as a series of hops from an adsorption site to another, which are mainly determined by the molecule-surface interaction potential, energy loss during hops, and temperature^17^,^25^. These factors are expected to be important for nanodroplets. However, the treatment of hopping transport for single molecules does not present the diffusion physics and may not work for nanodroplets. The diffusion of solid nanoparticles on surfaces is generally considered as a combination of rolling, sliding, and sticking motions^20^,^21^,^22^,^23^,^24^. Although studies show that liquid nanodroplets may behave like solid particles, which can roll and slide on surfaces^26^,^27^, nanodroplet diffusion on surfaces is more similar to the Brownian motion of colloidal nanoparticles^10^,^28^ in terms of diffusion mechanism because the friction at the droplet-surface interface plays a dominant role. Recently, some work shows that nanodroplets could diffuse very fast on vibrating graphene^29^ and carbon nanotube^30^ surfaces due to propagating ripples generated on the surfaces, where the shape of the droplets is a minor issue. On smooth surfaces, however, the shape change of droplets may greatly influence the diffusion. Moreover, the contact angle and contact area of droplets can be easily affected by external parameters, such as temperature and surface energy. These issues make the theoretical analysis challenging and leave nanodroplet surface diffusion poorly understood.

In this report, we investigate the diffusion of water nanodroplets on smooth surfaces through molecular dynamics (MD) simulations and theoretical analyses. The diffusion coefficient is calculated at different temperatures and surface wettabilities. The relationship between the diffusion coefficient *D* and temperature is found to depend on the surface wettability. The dependence of *D* on temperature changes from linear to nonlinear as the surface wettability is reduced. This is different from molecular surface diffusion. Furthermore, an expression for the diffusion coefficient is proposed, which is confirmed by MD simulations.

## Molecular Dynamics Simulations

**Figure 1:**
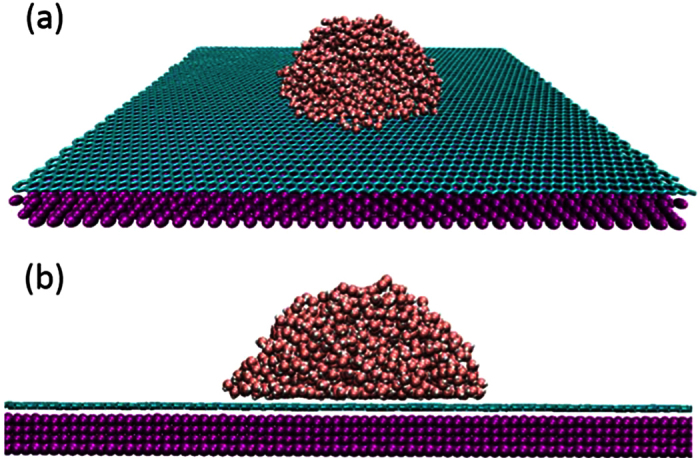
Snapshots of the MD simulation system (the surface is partially shown). Purple particles represent substrate atoms, on which a layer of graphene (cyan) is supported. The nanodroplet contains 1168 water molecules (H: white; O: red). (**a**) Perspective view. (**b**) Front view.

Molecular dynamics simulations are conducted using the LAMMPS package^31^. The simulation system consists of a water nanodroplet and a layer of graphene supported on a substrate, as illustrated in Fig. 1. The graphene is used to ensure a smooth surface. It is also of great interest as an emerging nanomaterial in the studies of droplet-surface interactions^23^,^32^. The SPC/E model^33^ and the AIREBO potential^34^ are employed to simulate water molecules and graphene, respectively. The SHAKE algorithm is used to maintain the 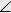
HOH angles and O–H bonds in water molecules. Each oxygen and hydrogen atom in water molecules carry a point charge *q* equal to −0.8476e and 0.4238e, respectively. The interaction between a pair of water molecules, *a* and *b*, is calculated using the combined Lennard-Jones (LJ) and Coulomb potential^35^,^36^


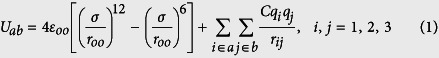


where 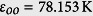 and 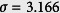 Å are the binding energy and collision diameter for oxygen atoms, 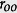 is the distance between the oxygen atoms of the two interacting water molecules, *C* is Coulomb’s constant, and the subscripts *i* and *j* represent the *i*^th^ and *j*^th^ atom (H or O) of water molecules *a* and *b*, respectively. The substrate is described by the embedded atom method potential and the parameters for silver are used^37^. The interactions among water, graphene, and the substrate are described by the LJ potential and the LJ parameters are obtained using the Lorentz-Berthelot mixing rule based on self-interacting parameters^35^,^38^. The LJ binding energy for graphene-water interaction is fixed as 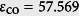 K, while that for droplet-substrate interaction 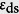 is varied to consider the effect of surface wettability.

Simulations are performed in canonical (*N, V, T*) ensembles, where the temperature is controlled by a Nosé-Hoover thermostat. The lengths of the simulation cell in the *x, y*, and *z* directions are 17.7, 19.6, and 20 nm, respectively. Periodic boundary conditions (PBCs) are applied in all the directions. Droplets containing 596 and 1168 water molecules are considered, for which the dimensions of the simulation cell are sufficiently large such that the interaction between the droplet and its periodic images caused by PBCs does not affect the motion of the droplet^39^. The cut-off distance is set to be 1.4 nm and 1 nm for the LJ and short range Coulomb potentials, respectively. Particle-Particle Particle-Mesh (PPPM) method is applied to account for the long range Coulombic force. The time step is set as 2 fs. There are four layers of atoms in the substrate. The atoms in the bottom layer of the substrate are fixed, while those in the other three layers are free to vibrate. The net force on graphene caused by the initialization is removed to keep graphene stationary. The system is relaxed for 300 ps, which is followed by data collection for at least 30 ns. Six simulations with different initial conditions are used to obtain the error bars for the diffusion coefficient.

## Result and Discussion

**Figure 2:**
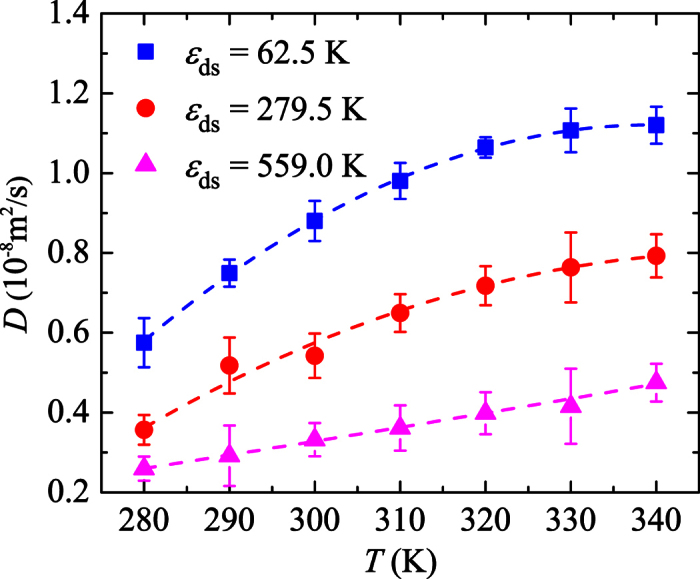
Diffusion coefficient of a nanodroplet containing 1168 water molecules as a function of temperature at different surface wettabilities. The dashed lines are polynomial fits to the data.

The diffusion of a nanodroplet containing 1168 water molecules (*n* = 1168) is studied first by changing the temperature *T* and droplet-substrate binding energy 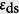, which governs the surface wettability. The diffusion coefficient *D* of the droplet in the *x*-*y* plane is obtained by calculating the mean square displacement (MSD) of the center-of-mass (COM) of the droplet,


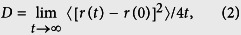


where *t* is time, 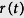 is the position of the COM of the droplet at time *t* in the *x*-*y* plane, and 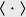 denotes the ensemble average. Figure 2 shows *D* as a function of *T* at different 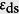 values in a wide range. It is seen that the dependence of *D* on *T* is greatly affected by the droplet-surface interaction strength. At large 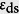 values, i.e. high surface energy and strong droplet-surface interaction, *D* increases linearly with increasing *T*. However, the dependence of *D* on *T* trends to be nonlinear as the interaction is weakened (the surface becomes less wetting), which is different from the surface diffusion of single molecules^17^.

The general explanation for the diffusion behaviors in Fig. 2 lies in the roles of temperature and 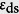, which measures the droplet-surface interaction strength. As 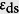 is increased, the friction at the interface is enhanced, which grows to be the dominant factor and reduces the diffusion coefficient at a given temperature, as indicated in Fig. 2. The effects of temperature are two folds. A high temperature on one hand enhances the mobility of the droplet, on the other hand, increases the friction between the droplet and surface due to the enhanced thermal motions of water molecules and substrate atoms, which strengthen the droplet-surface interaction, and the enlarged contact area when the temperature is raised, as will be discussed later. The contact area change is special for nanodroplets and makes the diffusion nontrivial. The linear increase of *D* in Fig. 2 for 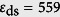 K is mainly due to the enhanced mobility of the droplet as *T* is increased because the friction caused by *T*, in this case, is insignificant compared with that due to the strong water-surface interaction. At small 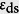 values, however, the droplet-surface interaction is relatively weak and the temperature induced friction is apt to be important, which counteracts the mobility enhancement as *T* is raised. This is why the diffusion coefficient levels off as *T* is increased.

**Figure 3:**
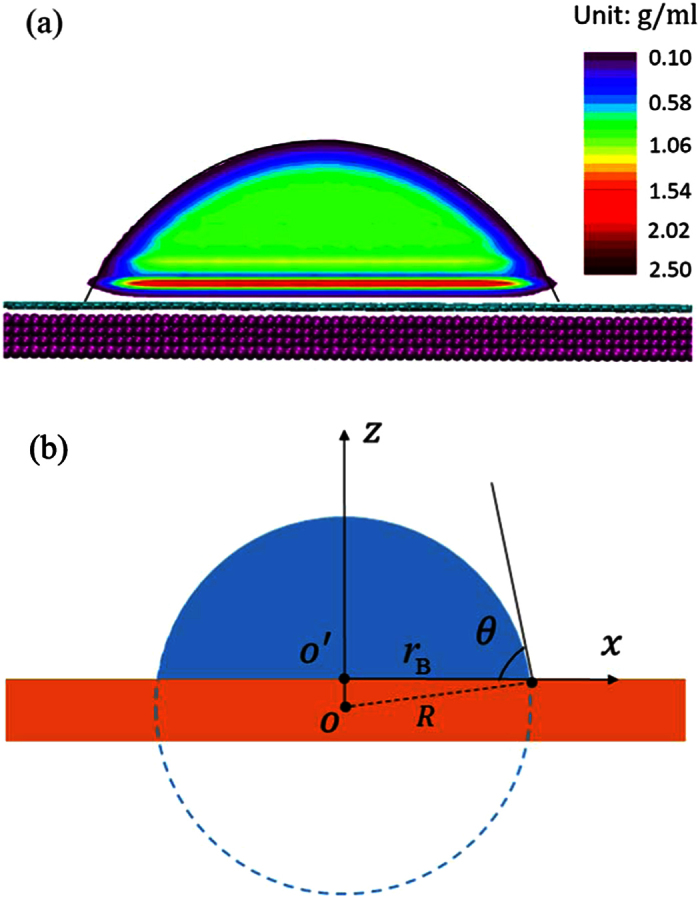
Determination of droplet contact angle and contact radius on solid surfaces. (**a**) Density contours of the droplet for 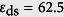 K and 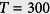 K. (**b**) Relationships among the contact angle *θ*, contact radius *r*_B_, and the radius of the sphere *R*.

**Figure 4:**
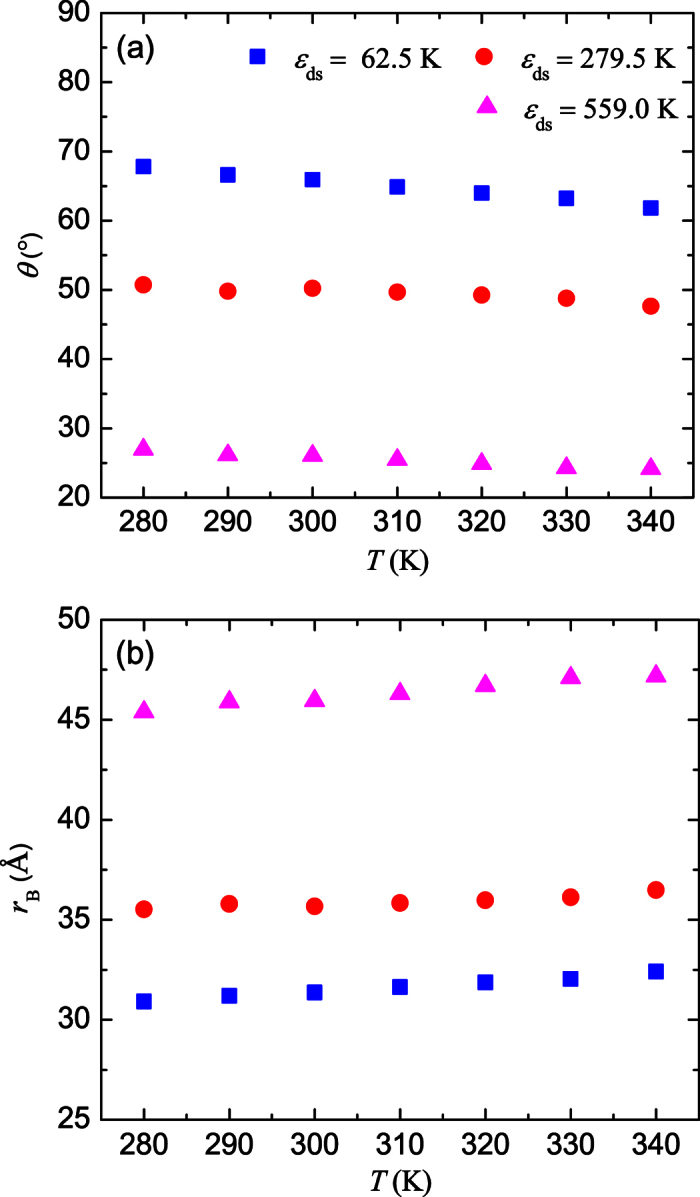


The coupled effects of temperature and droplet-surface interactions on the diffusion coefficient bring inconvenience in practical applications, where a simple expression for *D* is desired. To rephrase the results and develop a simple relation for *D*, it is preferred to employ the droplet contact angle *θ* and contact radius *r*_B_ (the radius of the contact area), which are two key parameters for describing droplet motions. The contact angle can be obtained based on the density distribution of the droplet at equilibrium. Figure 3(a) depicts the density contours of the droplet at room temperature, which can be used to determine the contact angle *θ* (Fig. 3(b)). It is also seen that the shape of the droplet can be approximated as a spherical cap^32^,^40^ and the contact radius *r*_B_ can be calculated as 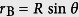 based on the radius of the sphere, 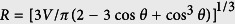
41, where *V* is the volume of the droplet, as illustrated in Fig. 3(b). Figure 4 shows *θ* and *r*_B_ of the droplet as a function of temperature at different 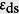 values. Generally, the contact angle decreases and the contact radius increases with increasing temperature for a given 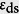 value. This is because the surface tension of water droplets decreases as temperature is raised, which reduces the contact angle according to the Young-Laplace equation^42^. At a constant temperature, the contact angle is reduced and the contact radius is increased as the droplet-surface interaction is strengthened because the surface becomes more wetting at larger 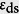 values^43^.

Theories for molecular surface diffusion and particle transport in fluids suggest that diffusion coefficients are mainly determined by *T* and friction coefficient *γ*^17^,^44^. To be in line with previous transport theories and for the ease of practical applications, a simple expression similar to Einstein’s relation is desired for nanodroplet diffusion if the friction coefficient could be properly defined,


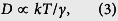


where *k* is the Boltzmann constant. According to the Green-Kubo correlation formula^45^,^46^,^47^, *γ* can be calculated as


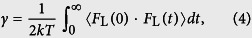


where 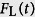 is the total lateral force acting on the droplet by the surface at time *t*. The force correlation term in Eq. (4) can be approximated as^46^


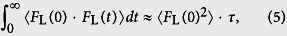


where *τ* is the relaxation time of the total lateral force, when 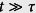, 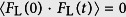. *τ* depends on the mean square force 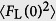 and is related to the relaxation of the surface tension, which is independent of the viscosity of the fluid^48^, indicating that *τ* is insensitive to temperature. Furthermore, we have calculated *τ* for different surface properties through MD simulations and found that *τ* remains unchanged even if the droplet-surface interaction is varied in a wide range, as shown in Table 1. Therefore, it is reasonable to treat *τ* as a constant. Generally, 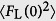 should be proportional to the contact area. However, for nanodroplets, 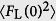 is mainly determined by the lateral force on the droplet around the contact line because the motions of water molecules at the interface are largely governed by the contact line and the surface tension close to the contact line is much larger than that at the interior part^48^. The mean area around the contact line is 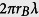, where *λ* is the radial fluctuation of the contact line and is roughly a constant. Hence, 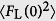 is proportional to the contact radius *r*_B_. In addition, 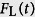 is also affected by temperature due to the thermal motion of water molecules and surface atoms and a linear dependence of 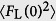 on temperature has been suggested for small droplets^49^. Considering that 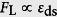, the friction coefficient *γ* in Eq. (4) can be written as


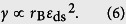


Droplet-surface LJ binding energy 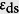 is a microscopic parameter, which is not suitable for characterizing droplet dynamics. Practically, contact angle *θ* is widely used for describing droplet motions and it is desired to represent 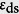 using *θ*. It has been shown that 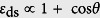
50,51,52, with which, it is obtained that


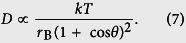


**Figure 5:**
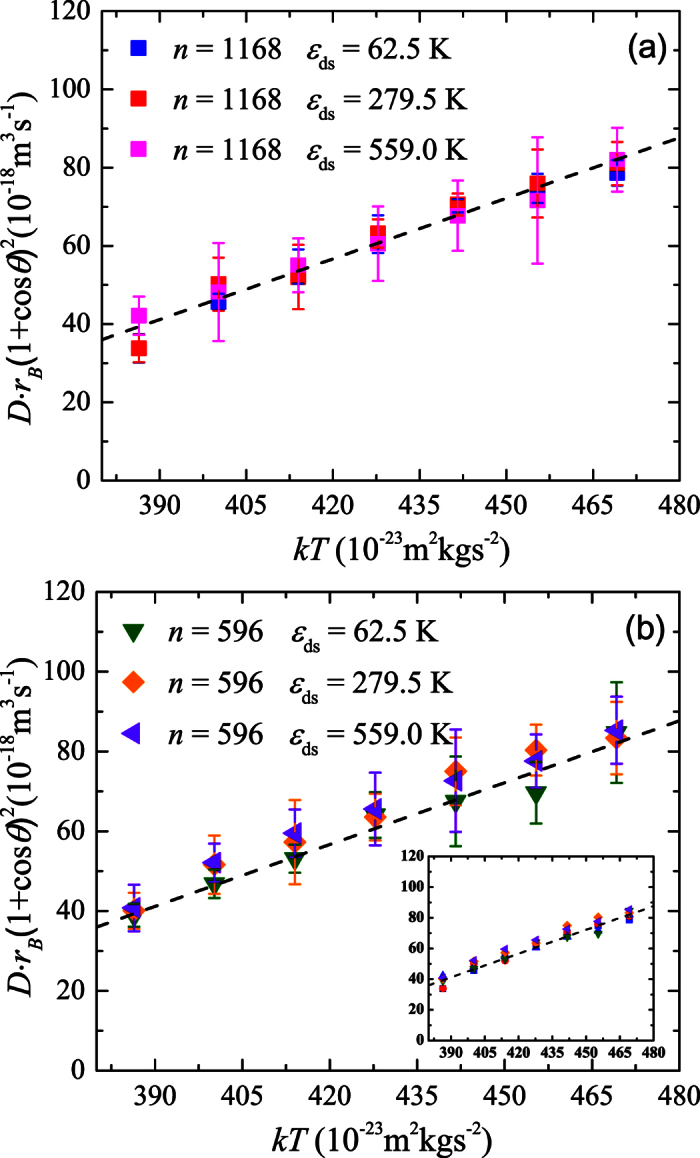
Diffusion coefficient of nanodroplets. (**a**) 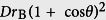 versus *kT* for the droplet containing 1168 water molecules. (**b**) 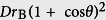 versus *kT* for the droplet containing 596 water molecules. The inset in (**b**) shows the linear fit for the diffusion coefficient of both droplets.

Equation (7) suggests a general relation for the surface diffusion of nanodroplets. Figure 5(a) plots 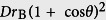 as a function of *kT* for the results in Fig. 2. It is seen that all the data roughly fall on the same straight line, which confirms the simple expression in Eq. (7). To further verify the Eq. (7), the diffusion coefficient of a droplet containing 596 water molecules is also calculated by changing the temperature for different surface wettabilities. As expected, the general dependence of *D* on *T* agrees very well with Eq. (7), as shown in Fig. 5(b). A linear fit for all the data in Fig. 5(a,b), as shown in the inset of Fig. 5(b), gives an expression for the diffusion coefficient


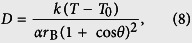


where 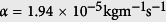 is a constant and 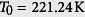. The unit of *α* is the same as viscosity. However, *α* is independent of droplet size and temperature. 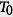 in Eq. (8) can be viewed as an ad hoc temperature, which might be related to the freezing point of water nanodroplets, below which droplets become solid particles and Eq. (8) is invalid.

It is noted that Eq. (8) is obtained by assuming that the fluctuations in the lateral force 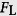 are mainly from molecules around the contact line, which is valid for small droplets. For sufficiently large droplets, the thermal motion of droplet molecules in the interior of the contact area may also contribute to the force correlation function. Hence, there might be a critical droplet size, beyond which Eq. (7) is inaccurate. This critical size is beyond the scope of current work and will be explored in the future. It is worth mentioning that 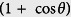 approaches zero as the contact angle is close to 180^o^, which is a singular point for Eq. (8). Actually, recent investigations^53^,^54^,^55^ showed that the relation, 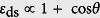, breaks down when the surface is superhydrophobic, where Eq. (7) is invalid.

## Conclusions

In summary, we have investigated the diffusion of water nanodroplets on supported graphene surfaces through MD simulations. A simple relation for the diffusion coefficient has been developed on the basis of theoretical analyses, which is confirmed by MD simulations.

## Additional Information

**How to cite this article**: Li, C. *et al*. A Relation for Nanodroplet Diffusion on Smooth Surfaces. *Sci. Rep.* **6**, 26488; doi: 10.1038/srep26488 (2016).

## Figures and Tables

**Table 1:** Relaxation time *τ* at different 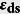 values at *T* = 280 K.

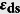 (K)	62.5	279.5	559.0
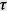 (fs)	89.08	89.41	91.67
